# Dense Collagen-I Matrices Enhance Pro-Tumorigenic Estrogen-Prolactin Crosstalk in MCF-7 and T47D Breast Cancer Cells

**DOI:** 10.1371/journal.pone.0116891

**Published:** 2015-01-21

**Authors:** Craig E. Barcus, Elizabeth C. Holt, Patricia J. Keely, Kevin W. Eliceiri, Linda A. Schuler

**Affiliations:** 1 Department of Comparative Biosciences, University of Wisconsin-Madison, Madison, Wisconsin, United States of America; 2 Cellular and Molecular Biology Program, University of Wisconsin-Madison, Madison, Wisconsin, United States of America; 3 Department of Cell and Regenerative Biology, University of Wisconsin-Madison, Madison, Wisconsin, United States of America; 4 Laboratory for Cellular and Molecular Biology and Laboratory for Optical and Computational Instrumentation, University of Wisconsin-Madison, Madison, Wisconsin, United States of America; 5 University of Wisconsin Paul P. Carbone Comprehensive Cancer Center, University of Wisconsin-Madison, Madison, Wisconsin, United States of America; University of California, San Diego, UNITED STATES OF AMERICA

## Abstract

Breast cancers that express estrogen receptor alpha (ERα+) constitute the majority of breast tumors. Estrogen is a major driver of their growth, and targeting ER-mediated signals is a largely successful primary therapeutic strategy. Nonetheless, ERα+ tumors also result in the most breast cancer mortalities. Other factors, including altered characteristics of the extracellular matrix such as density and orientation and consequences for estrogen crosstalk with other hormones such as prolactin (PRL), may contribute to these poor outcomes. Here we employed defined three dimensional low density/compliant and high density/stiff collagen-I matrices to investigate the effects on 17β-estradiol (E2) activity and PRL/E2 interactions in two well-characterized ERα+/PRLR+ luminal breast cancer cell lines *in vitro.* We demonstrate that matrix density modulated E2-induced transcripts, but did not alter the growth response. However, matrix density was a potent determinant of the behavioral outcomes of PRL/E2 crosstalk. High density/stiff matrices enhanced PRL/E2-induced growth mediated by increased activation of Src family kinases and insensitivity to the estrogen antagonist, 4-hydroxytamoxifen. It also permitted these hormones in combination to drive invasion and modify the alignment of collagen fibers. In contrast, low density/compliant matrices allowed modest if any cooperation between E2 and PRL to growth and did not permit hormone-induced invasion or collagen reorientation. Our studies demonstrate the power of matrix density to determine the outcomes of hormone actions and suggest that stiff matrices are potent collaborators of estrogen and PRL in progression of ERα+ breast cancer. Our evidence for bidirectional interactions between these hormones and the extracellular matrix provides novel insights into the regulation of the microenvironment of ERα+ breast cancer and suggests new therapeutic approaches.

## Introduction

Breast cancers that express estrogen receptor alpha (ERα^+^) constitute approximately 75% of all cases [1, 2]. Estrogen is a major driver of growth in these cancers, and targeting ER-mediated signals is a primary therapeutic strategy. While this is successful in many cases, approximately 25% of all ER^+^ tumors initially or eventually fail to respond to these treatments and result in poor clinical outcomes [3–6]. Despite our understanding of the mechanisms by which estrogen regulates transcription, we are only beginning to appreciate how estrogen activity is modulated by other factors in the tumor microenvironment. A major unstudied area is the changing properties of the extracellular matrix (ECM) and consequences for crosstalk with other hormones such as prolactin (PRL).

Advancing cancers elicit deposition of fibrillar collagens, known as desmoplasia [7]. This fibrotic response, which includes both increased collagen deposition and modified alignment, is well characterized in breast cancer, and is implicated in disease progression [8–12]. The increased mechanical stiffness leads to activation of signaling pathways including FAK and SRC-family kinases (SFK) that promote invasion and tumor progression [13–15]. Elevated collagen density reduces tumor latency and increases pulmonary metastases in the MMTV-PyMT murine model [16]. Clinically, collagen fibers oriented perpendicularly to the surface of ERα+ tumors identified patients with a 3-fold increased relative risk for poor outcomes [10]. However, the effects of these changes in the ECM on estrogen actions have not been examined.

High circulating PRL is a risk factor for metastatic ERα^+^ breast cancer [17, 18], and its cognate receptor (PRLR) is expressed in most breast cancers, especially those expressing ERα [19, 20]. PRL has been shown to cooperate with estrogen in 2-dimensional cultures of breast cancer cell lines. In these systems, PRL enhances estrogen-induced growth of T47D and MCF-7 breast cancer cells [21–24], augments estrogen-regulated transcriptional activity, and prolongs signaling [20, 24–26]. Moreover, PRL and estrogen cross-regulate expression of each other’s receptors [27–29]. These hormones together stimulated budding of T47D colonies in three dimensional (3D) collagen matrices of physiologic stiffness [30], but the consequences of increased ECM stiffness were not examined.

PRL binding to PRLR initiates signaling cascades through multiple down-stream partners, including Janus kinase 2 (JAK2) and SRC family kinases (SFKs) [31–34]. Most physiological PRL actions on the mammary gland are mediated through the JAK2/STAT5 pathway [35], and in breast cancer, activated STAT5 predicts sensitivity to estrogen targeted therapies and favorable clinical outcomes [36–38]. However, PRL-activated SFKs mediate pro-tumorigenic signals and proliferation in breast cancer cell lines cultured on plastic [33, 34]. Using 3D culture in collagen-I matrices, we previously demonstrated marked effects of ECM stiffness on the spectrum of PRL-induced signals and behavioral outcomes in luminal breast cancer cells [39]. In compliant matrices, PRL activates STAT5 and stimulates development of well-differentiated colonies. In contrast, stiff matrices strengthen PRL signals to FAK-SFK-ERK1/2, increasing MMP-2 synthesis and activity and invasive behavior, and driving development of disorganized colonies. Under these conditions, PRL induces collagen reorganization, increasing the incidence of radially oriented fibers, as found in invasive clinical carcinomas [10]. These observations raise important questions regarding the effect of matrix density on estrogen action, and the interplay between PRL and estrogen in breast cancers surrounded by desmoplastic stroma.

Here we examined the effect of matrix density on 17β-estradiol (E2) activity and PRL/E2 interactions in two well-characterized, ERα^+^, PRLR^+^, luminal breast cancer cell lines cultured in defined 3D compliant and stiff collagen-I matrices. We report that matrix density modulated E2-induced transcripts, but did not alter the growth response. However, ECM density was a potent determinant of the behavioral outcomes of estrogen and PRL crosstalk. High density/ stiff, but not low density/ compliant matrices enhanced PRL/E2-induced growth mediated by increased activation of SFKs, and reduced responsiveness to the estrogen antagonist, 4-hydroxytamoxifen (4-OHT). It also permitted the combination of these hormones to drive invasion and modify the alignment of collagen fibers. Our studies demonstrate the power of matrix density to regulate the outcomes of hormone actions, and identify high density/ stiff matrices as critical collaborators of estrogen and PRL to drive progression of ERα+ breast cancer.

## Materials and Methods

### Reagents

17β-estradiol (E2) was purchased from Sigma-Aldrich (St. Louis, MO). Recombinant hPRL (Lot AFP795) was obtained from Dr. A.F. Parlow (National Hormone and Pituitary Program, NIDDK, National Institutes of Health, Torrance, CA). Type-I rat tail collagen (#CB354249) was obtained from Fisher Scientific (Pittsburgh, PA). Inhibitors used for these studies were purchased as follows: 4-hydroxy-tamoxifen (4-OHT) (#579002) from EMD Millipore (Billerica, MA), SFK inhibitor, PP-1 (#EI275) from Biomol International, LP (Plymouth Meeting, PA), and JAK2 inhibitor, BMS-911543 (#CT-BMS91) from Chemietek (Indianapolis, IN). Type-I collagenase (#17100–017) was purchased from Invitrogen (Grand Island, NY). Antibodies used in these studies were as follows: PRLR-ECD (#35–9200) and pSRC Y418 (#44660G) from Invitrogen (Grand Island, NY); ERK1/2 (#9102) and cleaved caspase-3 (#9661) from Cell Signaling Technology (Danvers, MA); cSRC (sc-18) and EGFR (sc-03) from Santa Cruz Biotechnology (Santa Cruz, CA); ERα (#NCL-ER-6F211/2) from Novocastra (Newcastle, United Kingdom); pan-actin (#125-ACT) from Phosphosolutions (Aurora, CO); Ki-67 (#AB15580) from Abcam (Cambridge, MA). Multiwell non-tissue culture-treated plates were obtained from Corning Life Sciences (#08–772–49 and #08–772–51, Tewksbury, MA). All other reagents were obtained from Fisher Scientific or Sigma-Aldrich.

### Cell culture

ERα^+^, PRLR^+^ T47D [40] and MCF-7 [41] breast cancer cells, as well as stable ERE-luciferase expressing clones (T47D-KBluc [42] and MELN respectively [43]), were maintained as previously described [41, 44]. Cells were cultured in phenol-red free RPMI 1640 supplemented with 5% charcoal-stripped fetal bovine serum (CSS) for 72h prior to plating in three dimensional collagen cultures. T47D and MCF-7 cells were plated in low density (LD)/ compliant (1.2 mg/ml) or high density (HD)/ stiff (2.8 mg/ml) type-I rat tail collagen in non-tissue culture treated multi-well plates as previously described [39, 40]. These concentrations were empirically derived for each cell line: “compliant” collagen gels can be contracted by cells over time, and “stiff” gels resist contraction [40, 47]. We and others have shown that 1.2 mg/ml collagen-I gels have an elastic modulus of approximately 13kPa, while 2.8 mg/ml collagen-I gels have an elastic modulus of approximately 23 kPa, as measured by tensile testing [45, 46]. The shear moduli for 1.2 mg/ml and 2.8 mg/ml collagen-I are approximately 0.1 kPa and 0.4 kPa, respectively, as measured by controlled strain rheometry [47]. After 24 h, the gels were released in phenol red free serum free (24 h experiments) or 5% CSS (72h-7d experiments). For 24h treatment experiments, cells were serum starved overnight prior to hormone treatments. For longer experiments, treatments were begun immediately after releasing the gels. Final hormone concentrations were 1nM E2 and/or 4nM PRL unless otherwise specified.

### ERE-luciferase activity

T47D-KBluc and MELN cells were plated in low density/ compliant (1.2 mg/ml) or high density/ stiff (2.8 mg/ml) type-I collagen gels as described above. Cultures were treated with vehicle (EtOH, 1:1000) or E2 (1nM) for 24h and cell lysates were analyzed for luciferase reporter activity as previously described [48], except that relative luciferase activity was normalized to total protein.

### Immunoblotting and quantitative Real-Time PCR

Immunoblotting and qRT-PCR were performed as previously described [39]. Immunoblot signals were visualized using enhanced chemiluminscence (ThermoFischer), and quantified by scanning densitometry (VisionWorksLS, v7.1, UVP, Upland, CA). qRT-PCR data was analyzed via the delta-delta C(t) method to 18S ribosomal RNA. Primer sequences for endogenous estrogen target genes analyzed can be found in S1 Table.

### Invasion assay

Invasion assays were performed as previously described [39]. Briefly, MCF-7 cells (3×10^5^/well) were mixed with appropriate concentrations of type I collagen to yield compliant or stiff matrices and vehicle/ hormones [EtOH (1:1000), EtOH + PRL (8nM), E2 (2nM), or E2 + PRL]. The cell/collagen mixture (300 µl) was plated onto transwell permeable supports (12 well, 8-µm pores; BD Biosciences, San Jose, CA) and allowed to polymerize for 30 min at room temperature. Phenol red free RPMI 1640 medium containing 10% gelding-horse serum was placed in the lower chamber and the system was incubated at 37°C for 24 h. Traversed cells were stained with Giemsa, and quantified.

### Cell growth assay

Cell growth was measured by changes in cell number. Briefly, T47D (1.2×10^5^ cells) or MCF-7 (2×10^4^ cells) were plated in compliant or stiff type-I collagen in 24-well non-tissue culture plates (200 µl total mixture) and allowed to polymerize as described above. After releasing the gels, 800 µl phenol red-free RPMI 1640 + 5% CSS was added to each well with treatments as indicated. MCF-7 experiments were harvested after 72 h. T47D cells were allowed to grow for 7d, and half of the media was exchanged with fresh hormone(s) every 72 h. Experiments utilizing inhibitors were pretreated for 1 h with vehicle (DMSO 1:1000 or EtOH 1:1000), ER antagonist 4-OHT (100 nM), SFK inhibitor PP-1 (500 nM), or JAK2 inhibitor BMS-911543 (125 nM [49]), prior to addition of hormones. At the end of the experiment, the gels were digested with 0.125% type-I collagenase in PBS for 30 min at 37°C. The solution was centrifuged at 250 x g for 5 min and the supernatant replaced with 2% paraformaldehyde to fix cells in single cell suspension, and cells were counted using a hemocytometer.

### Immunofluorescence

Immunofluorescence was performed as described [40], except that cells were permeabilized in 0.1% Triton X-100. Primary antibodies for Ki-67 and cleaved caspase-3 were added to the gels for 1h at room temperature, followed by extensive washing in PBS with 0.1% Tween-20 (PBS-T) and subsequent secondary antibody addition (anti-rabbit TRITC) and 4’,6-diamidino-2-phenylindole (DAPI) to stain nuclei. Fluorescent images were obtained on an E600 Eclipse fluorescence microscope with an RGB camera and Nikon NIS-Elements imaging software. 50–100 cells per field were counted in triplicate for each treatment and images were analyzed utilizing the NIH ImageJ software [50].

### Multiphoton microscopy, second harmonic generation, and collagen alignment quantification

Multiphoton microscopy and second harmonic generation imaging (20x objective) of collagen (890 nm, no filter) and NADH (780 nm, 460 ± 80nm filter) was performed at the Laboratory for Optical and Computational Imaging (LOCI) as previously described [39, 51]. Quantification of the mean relative angle of collagen within 50 µm of the cell membrane was performed utilizing the LOCI developed software CurveAlign as described [52].

### Statistical analyses

Statistical analyses were performed using GraphPad Prism v.4.0. Independent experiments were analyzed via one-way ANOVA with the Tukey comparison post-test or unpaired t-test for collagen alignment experiments. Significance was defined as a p-value less than 0.05.

## Results

### High density collagen matrices decrease ERE-activity in response to estrogen

**Figure 1:**
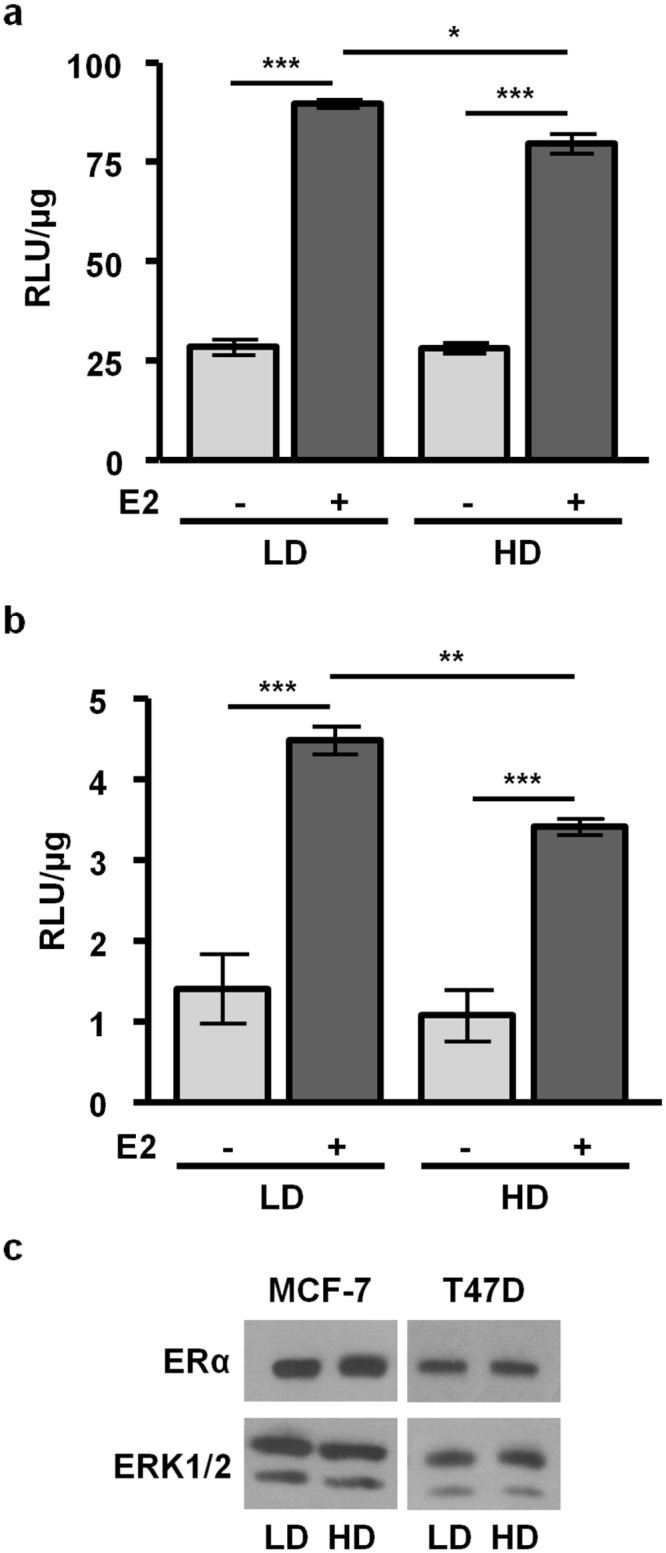
High density/ stiff collagen matrices decrease E2-induced ERE-luciferase expression in MCF-7 and T47D cells. **a)** MCF-7 cells stably transfected with an ERE-luciferase construct (MELN) were plated in low density (LD) or high density (HD) collagen and treated ± 1nM E2 for 24h, and luciferase activity determined as described in the Materials and Methods. n = 3, *p<0.05, ***p<0.001. **b)** T47D cells stably transfected with an ERE-luciferase construct (T47D-KBluc) were treated and harvested as in (a). n = 3, **p<0.01, ***p<0.001. **c)** Matrix density did not affect ERα expression. Lysates from cells plated in LD or HD collagen were examined for ERα expression. Representative blots shown.

Estrogen regulates multiple transcriptional enhancers, including the canonical estrogen responsive element (ERE). In order to investigate the effect of matrix density on estrogen regulated promoter activity, we cultured MELN or T47D-KBluc cells in low density/ compliant or high density/ stiff collagen-I matrices and treated +/- 17β-estradiol (E2) for 24 h. E2 robustly induced ERE-activity in both compliant and stiff matrices compared to vehicle controls (p<0.001). However, stiff matrices reduced E2-induced ERE activity compared to compliant matrices in both MCF-7 (p<0.05) (Fig. 1a) and T47D cells (p<0.01) (Fig. 1b), without altering ERα protein levels (Fig. 1c).

### Matrix density alters E2-induced gene transcription in a cell context dependent manner

**Figure 2:**
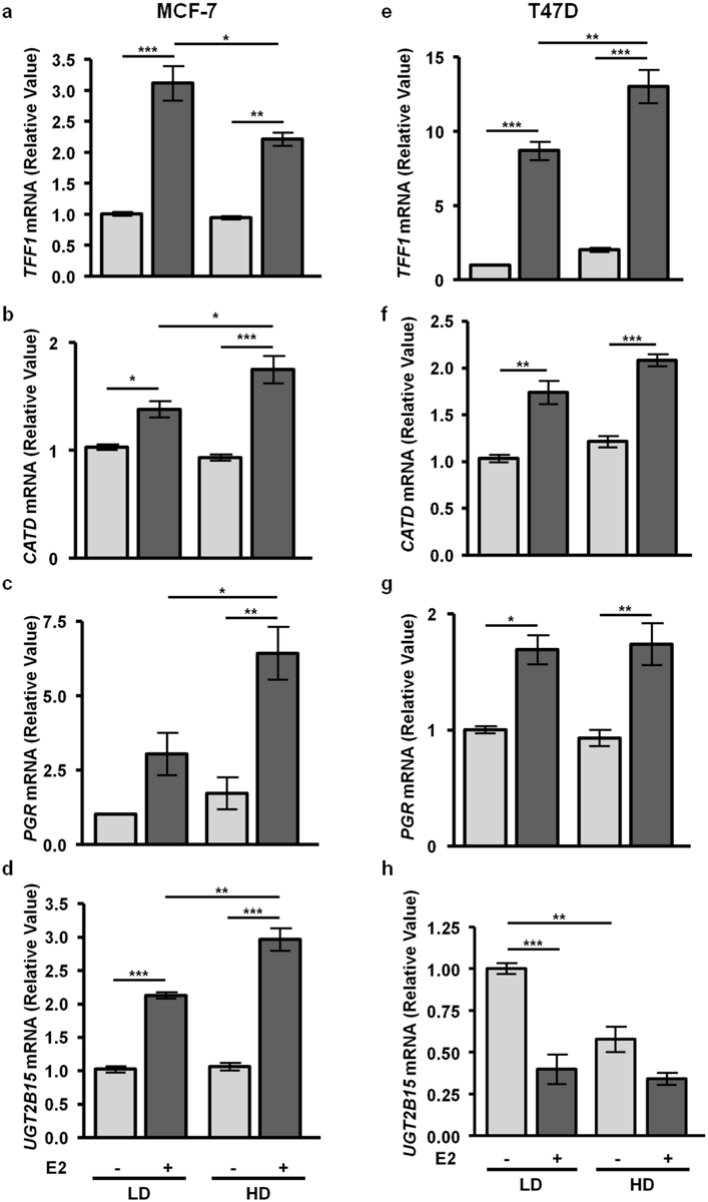
Matrix density alters E2-induced changes in transcripts in MCF-7 and T47D cells in a cell-context dependent manner. **a–d)** MCF-7 cells were cultured in LD or HD collagen, treated ± 1nM E2 for 24h, and RNA was harvested and mRNA was analyzed via qRT-PCR for the estrogen target genes, *TFF1* **(a)**, *CATD* **(b)**, *PGR* **(c)**, and *UGT2B15* **(d)**. **e-h)** T47D cells were cultured and analyzed as above for the estrogen-target genes, *TFF1* **(e)**, *CATD* **(f)**, *PGR* **(g)**, and *UGT2B15* **(h)**. n = 3, *p<0.05, **p<0.01, ***p<0.001.

Estrogen regulates transcription of many of its target genes by multiple enhancers, including not only EREs, but also AP-1, NFκB and Sp1 responsive enhancers via tethered ERα actions [53]. In order to determine if matrix density alters E2-induced transcriptional responses, we cultured T47D and MCF-7 cells in low density/ compliant or high density/ stiff collagen-I matrices and treated +/- E2 for 24h and examined transcripts of several well-studied endogenous target genes via qRT-PCR. In MCF-7 cells, stiff matrices reduced the ability of E2 to induce *TFF1* transcripts compared to compliant matrices (Fig. 2a), but increased E2-induced transcripts for *CATD* (Fig. 2b), *PGR* (Fig. 2c), and the AP-1 responsive gene, *UGT2B15* [54] (Fig. 2d) (p<0.05). In T47D cells, stiff matrices increased E2-induced *TFF1* transcripts (Fig. 2e) (p<0.01), but had no significant effect on *CATD* (Fig. 2f) and *PGR* mRNAs (Fig. 2g). In contrast to MCF-7 cells, unstimulated levels of *UGT2B15* mRNA in T47D cells were strongly decreased in stiff matrices (p<0.01), and further decreased by E2-treatment in compliant matrices (p<0.001) (Fig. 2h). The decreased AP-1-dependent gene transcription in T47D cells can be attributed to the higher expression of repressive AP-1 components in these cells [44].

### Estrogen induces growth of MCF-7 and T47D cells independent of matrix density

**Figure 3:**
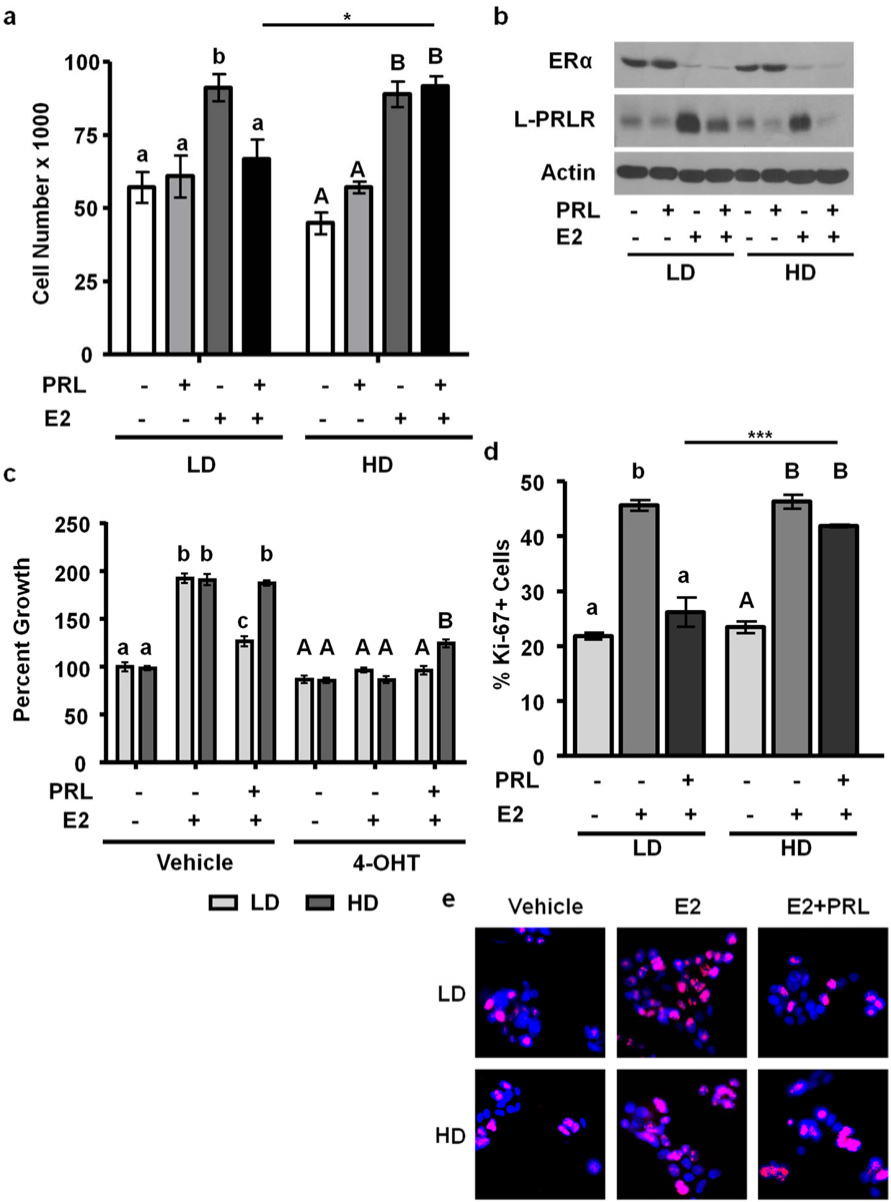
E2 and PRL co-treatment inhibits MCF-7 cell growth and proliferation in low density/ compliant but not high density/ stiff collagen matrices. **a)** MCF-7 cells were cultured in LD or HD collagen and treated ± E2, ± PRL for 72h. Cells were removed from gels and counted. n = 3. **b)** MCF-7 cells were cultured and treated as in **(a)** and lysates were immunoblotted with the indicated antibodies. Representative blots shown. **c)** MCF-7 cells were cultured as in **(a)** and treated ± 4-OHT 1h prior to hormone treatment. n = 4. **d,e)** MCF-7 cells were cultured and treated as in **(a)**. Gels were fixed and stained for DAPI and Ki-67, and quantified as described in the Materials and Methods (d). n = 3. **e)** Representative images of DAPI/ Ki-67 are shown in (d). Different letters represent significant differences (p<0.05) among treatments within each matrix condition **(a,d)** (LD, lower case; HD, upper case) or inhibitor treatment **(d)** (vehicle, lower case; 4-OHT, upper case). Asterisks indicate significant differences between the same treatments in different collagen densities, *p<0.05; ***p<0.001.

**Figure 4:**
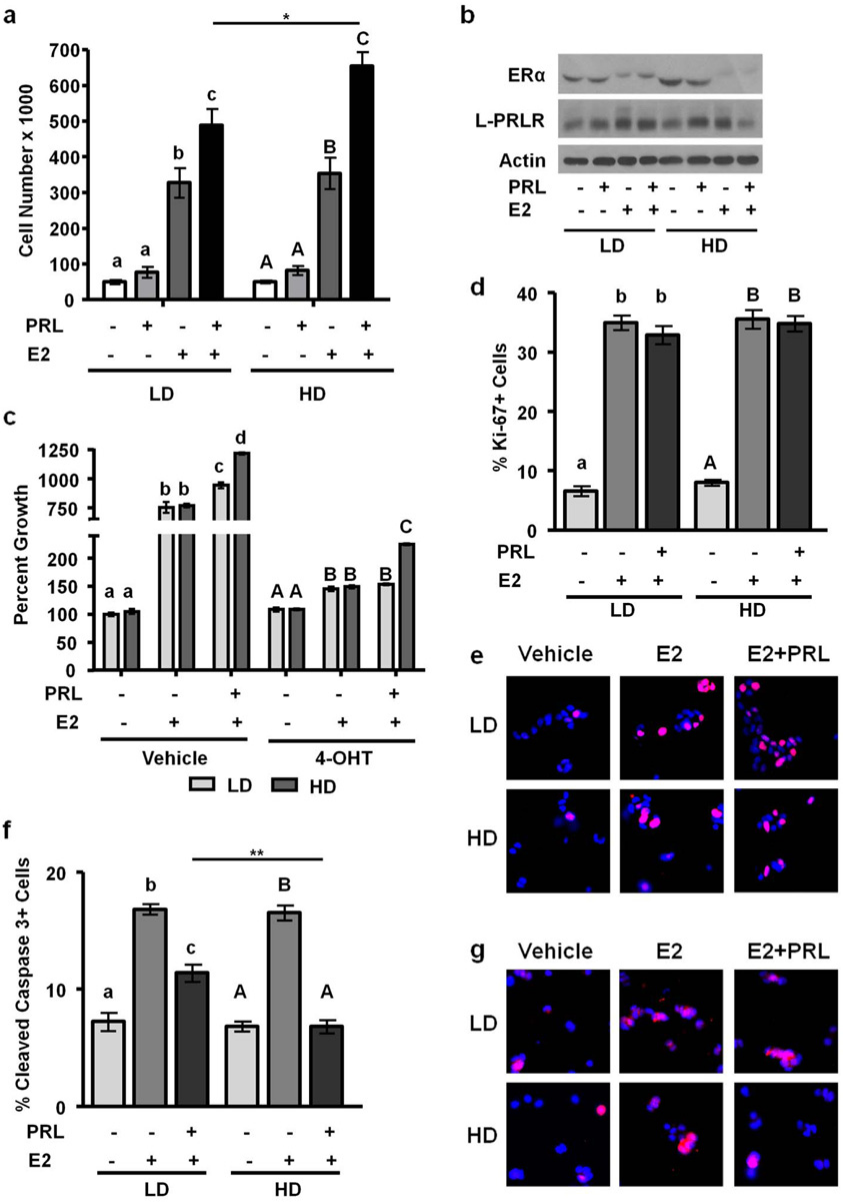
High density/ stiff collagen matrices enhance E2 and PRL induced cell growth, and inhibit apoptosis in T47D cells. **a)** T47D cells were cultured in LD or HD collagen and treated ± 1nM E2, ± 4nM PRL for 7d. Cells were removed from gels and counted via hemocytometer. n = 4. **b)** T47D cells were cultured in LD or HD collagen and treated ± 1nM E2, ± 4nM PRL for 72h and lysates were immunoblotted with the indicated antibodies. **c)** T47D cells were cultured as in **(a)**, and treated ± 4-OHT 1h prior to hormone treatments and quantitation of cell number. n = 4. **d,e,f,g)** T47D cells were cultured as in **(b)**, fixed and stained for DAPI and Ki-67 **(d,e)** or cleaved caspase 3 **(f,g)**, and quantified as described in the Materials and Methods. n = 3. Representative images of DAPI/ Ki67 are shown in (e) and DAPI/ cleaved caspase 3 are shown in (g). Different letters represent significant differences (p<0.05) among treatments within each matrix condition **(a,d,f)** (LD, lower case; HD, upper case) or inhibitor treatments **(c)** (vehicle, lower case; 4-OHT, upper case). Asterisks indicate significant differences between the same treatments in different densities, *p<0.05, **p<0.01.

Estrogen is a potent mitogen for these luminal breast cancer cell lines, but the effect of matrix density on this activity has not been reported. We therefore cultured these cell lines +/- E2 in low density/ compliant or high density/ stiff collagen-I gels for 72 h (MCF-7 cells) or 7 days (T47D cells). E2 treatment alone significantly increased cell number, as expected. However, matrix density had no effect on E2-induced growth (Figs. 3a, 4a), in contrast to its effects on E2-regulated transcripts. Moreover, matrix density did not alter the ability of 4-OHT to inhibit E2-stimulated growth.

### Matrix density controls the response to E2 and PRL co-treatment

Estrogen crosstalk with hormones is a well-recognized feature of breast biology, yet the effect of matrix density on these interactions is poorly understood. To elucidate the effect of matrix density on estrogen and PRL crosstalk, we first examined the net effect on expression of ERα and the long isoform of the PRLR (L-PRLR) by western analyses. After 72h of hormone treatment, E2 had strikingly down-regulated ERα in both cell lines regardless of matrix density (Figs. 3b, 4b). PRL did not strongly alter steady state ERα levels under these conditions. The effects on PRLR levels were more complex. E2 raised PRLR, most markedly in MCF-7 cells, consistent with the transcriptional activation previously reported [28], independent of matrix density. PRL down-regulated its receptors in MCF-7 cells as previously reported [55], regardless of matrix density (Fig. 3b). However, PRL modestly up-regulated L-PRLR in T47D cells, consistent with reported effects in some other target tissues, and complex transcriptional and post-transcriptional regulation [56] (Fig. 4b). Interestingly, E2 and PRL in combination down-regulated L-PRLR in high density/ stiff collagen-I matrices more than low density/ compliant matrices, indicating that matrix density alters the outcome of estrogen and PRL crosstalk to PRLR (Figs. 3b, 4b).

In contrast to the modest PRL-induced increase in growth of breast cancer cells cultured on tissue culture plastic [34, 41], PRL alone did not significantly increase growth of either MCF-7 or T47D cells in the 3D collagen-I matrices. However, combinatorial E2 and PRL treatment altered growth in a matrix- dependent and cell line specific manner. In MCF-7 cells cultured in low density/ compliant matrices, PRL decreased cell number compared with E2 alone (p<0.05, Fig. 3a). In high density/ stiff matrices, this inhibitory effect of PRL on E2-induced growth was relieved (p<0.05). Furthermore, cells treated with E2 and PRL grew modestly but significantly in the presence of the ERα antagonist 4-OHT only in stiff matrices (p<0.05, Fig. 3c). Rates of proliferation examined by Ki67 staining in response to E2 and PRL together exhibited a pattern similar to cell growth (Fig. 3d).

High density/ stiff matrices also enhanced PRL and E2 crosstalk to increase growth of T47D cells, but the outcome and underlying processes differed. In contrast to MCF-7 cells, E2 and PRL co-treatment significantly increased cell number in both low density/ compliant and high density/ stiff matrices, compared to E2 alone (p<0.05, Fig. 4a). This response was significantly enhanced in stiff matrices (p<0.05) (Fig. 4a). Like in MCF-7 cells, E2 and PRL co-treatment significantly increased growth in the presence of 4-OHT especially in stiff matrices (p<0.05), where it doubled growth compared to vehicle treated cultures (p<0.05, Fig. 4c). However, in contrast to MCF-7 cells, augmented proliferation did not underlie PRL interaction with E2 (Fig. 4d). Rather, the ability of PRL to repress E2-induced apoptosis was increased in stiff matrices (Fig. 4e). PRL is a known anti-apoptotic factor on tissue culture plastic [57–59], and these data indicate stiff matrices enhance the pro-survival effect of PRL on T47D cells.

### JAK2 mediates PRL-enhanced growth regardless of matrix density

**Figure 5:**
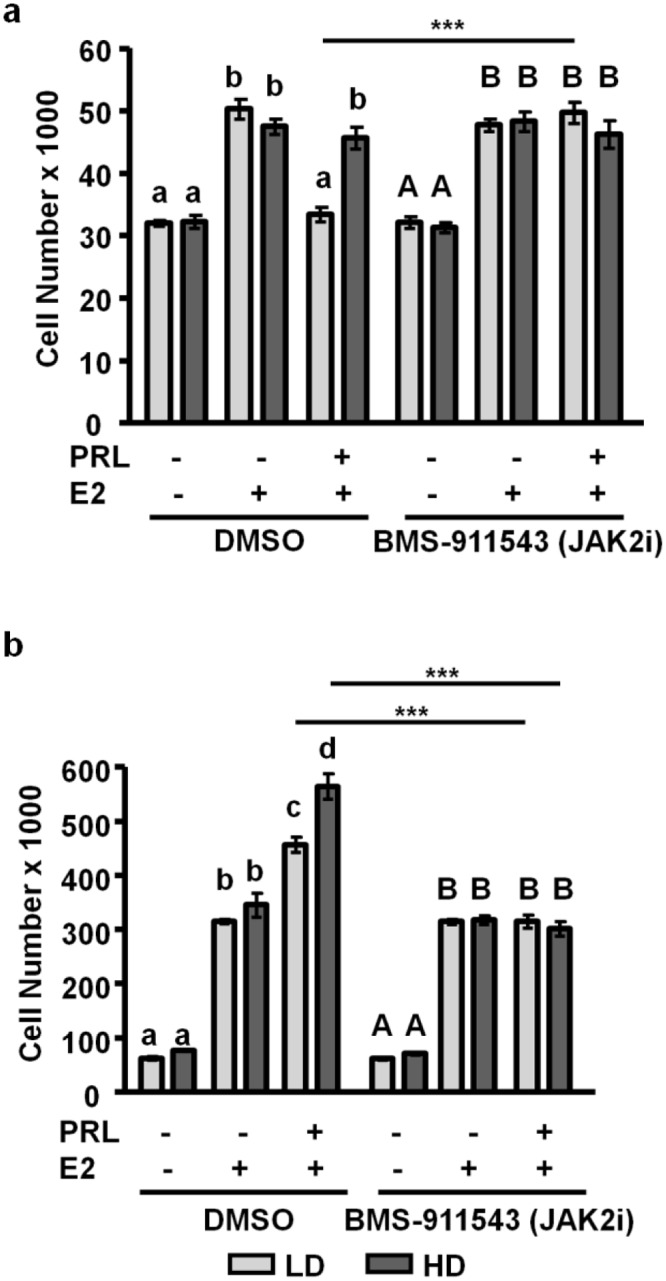
JAK2 mediates PRL-driven growth regardless of matrix density. **a)** MCF-7 cells were cultured in LD or HD collagen-1 gels, pretreated for 1h with vehicle or 125 nM of the Jak2 inhibitor, BMS-911543, and then cultured for an additional 72h with the indicated hormones, and cells were counted. n = 3. **b)** T47D cells were cultured in LD or HD collagen gels, pretreated for 1h with vehicle or 125 nM BMS-911543, and then cultured for an additional 7d with the indicated hormones, and cells were counted. n = 3. Different letters represent significant differences (p<0.05) within each inhibitor treatment (vehicle, lower case; JAK2 inhibitor (BMS-911543), upper case). Asterisks indicate significant differences between the same treatments in different densities, ***p<0.001.

PRL activates downstream signaling cascades through proximal kinases, including JAK2 and SFKs [31–34]. We have previously shown that low density matrices favor PRL-initiated JAK2-STAT5 signals, compared to high density matrices [39]. In order to determine the contributions of JAK2 to PRL-enhanced E2-stimulated growth, we cultured MCF-7 and T47D cells in low density/ compliant or high density/ stiff collagen-I matrices and treated with either DMSO or the JAK2 small molecule inhibitor, BMS-911543, prior to hormone treatments. BMS-911543 did not affect either vehicle or E2 treatment alone in either cell line or matrix condition. In MCF-7 cells, inhibiting JAK2 increased E2+PRL induced growth in low density matrices compared to control treatment (p<0.001), while growth in high density matrices was not affected (Fig. 5a). In T47D cells, inhibiting JAK2 abrogated all PRL-induced growth regardless of matrix density (p<0.001), reducing growth to that of E2 treatment alone (Fig. 5b). These results indicate that in MCF-7 cells, JAK2 is a key mediator of PRL crosstalk with E2 to growth only in low density matrices; while in T47D cells, JAK2 is necessary for PRL-effects on E2-induced growth, regardless of matrix density.

### SFKs control PRL-induced cell growth only in high density/ stiff collagen matrices

**Figure 6:**
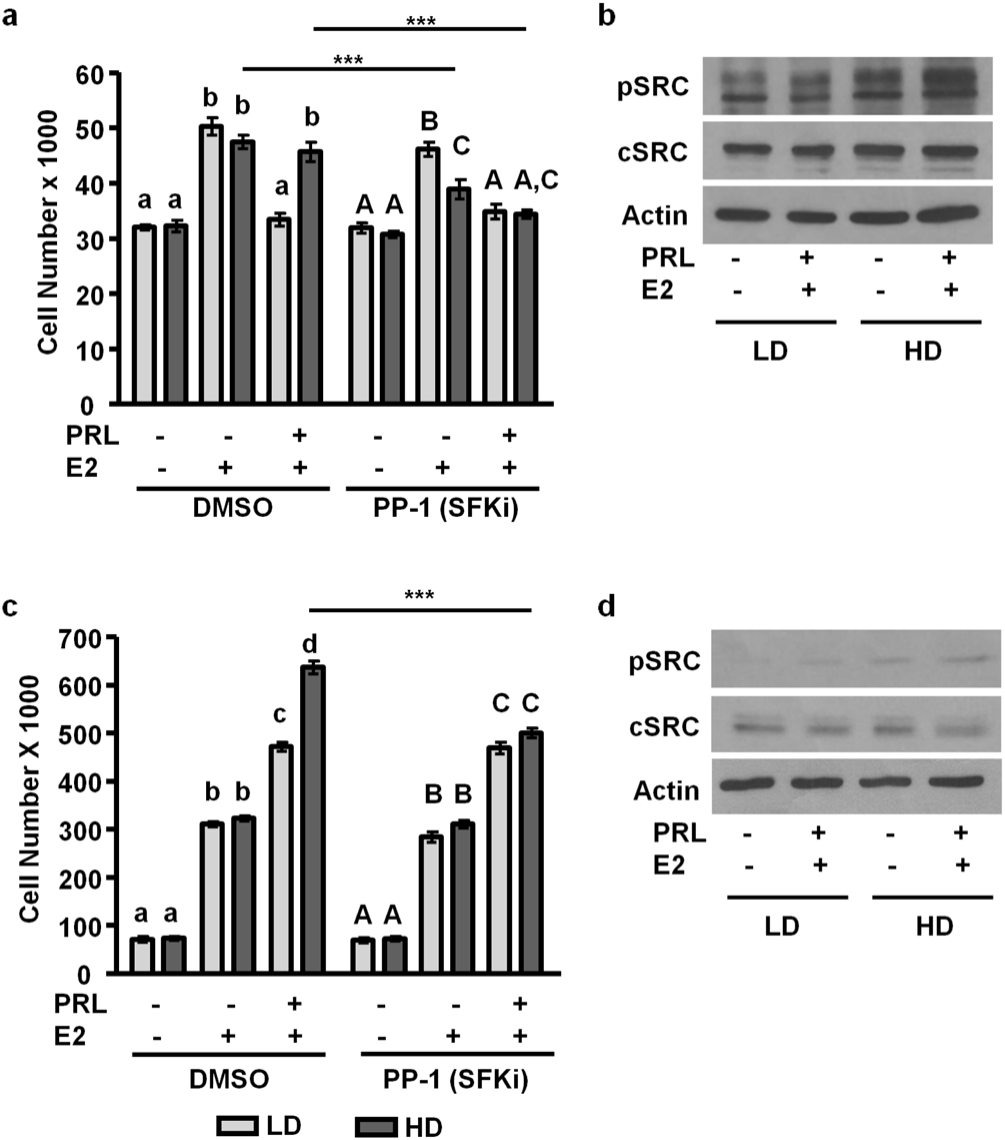
SFKs mediate PRL-augmented growth only in high density/ stiff collagen matrices. **a)** MCF-7 cells were cultured in LD or HD collagen-1 gels, pretreated for 1h with vehicle or 500nM of the SFK inhibitor, PP1, and then cultured for an additional 72h with the indicated hormones, when cells were counted. n = 3. **b)** MCF-7 cells were cultured in LD or HD collagen gels, treated for 10min as indicated, and lysates were immunoblotted with the indicated antibodies. Representative blots shown. **c)** T47D cells were cultured in LD or HD collagen gels, pretreated for 1h with vehicle or 500nM of the SFK inhibitor, PP1, and then cultured for an additional 7d with the indicated hormones, when cells were counted. n = 3. **d)** T47D cells were cultured in LD or HD collagen gels, treated for 10min as indicated, and lysates were immunoblotted with the indicated antibodies. Representative blots shown. Different letters represent significant differences (p<0.05) within vehicle or PP1 treatments. Asterisks indicate significant differences between the same treatments in different densities, ***p<0.001.

While the JAK2/STAT5 cascade is the major physiological pathway for PRL actions in mammary function [35], SFKs mediate many PRL signals in breast cancer [33, 34]. Additionally, SFKs play a role in extra-nuclear ERα-mediated signals [60, 61]. In order to determine the role of SFKs in E2 and PRL-induced cell growth, we cultured both MCF-7 and T47D cells as above and treated with either DMSO vehicle or the SFK inhibitor, PP-1. In MCF-7 cells, inhibiting SFKs significantly reduced E2-induced cell growth in stiff collagen matrices, but not compliant matrices (p<0.05), and completely blocked PRL-enhanced E2-induced growth in stiff collagen matrices (p<0.001) (Fig. 6a). MCF-7 cells treated for 10min with E2 and PRL show increased SFK phosphorylation in stiff matrices compared to compliant matrices (Fig. 6b). In T47D cells, SFK inhibition had no effect on cell growth in compliant matrices, but reduced PRL-enhanced E2-induced growth in stiff collagen matrices to levels comparable to compliant matrices treated with E2 and PRL (p<0.001) (Fig. 6c). Like MCF-7 cells, T47D cells responded to E2 and PRL with increased SFK phosphorylation in stiff matrices (Fig. 6d). Decreased cSRC levels in stiff matrices have been correlated with increased activation and turnover via ubiquitin-mediated proteasomal degradation [62, 63]. These results indicate that SFKs play a role in E2/PRL crosstalk to growth only in stiff collagen matrices.

### E2 and PRL co-treatment induces invasion and collagen realignment of MCF-7 cells in high density collagen matrices

**Figure 7:**
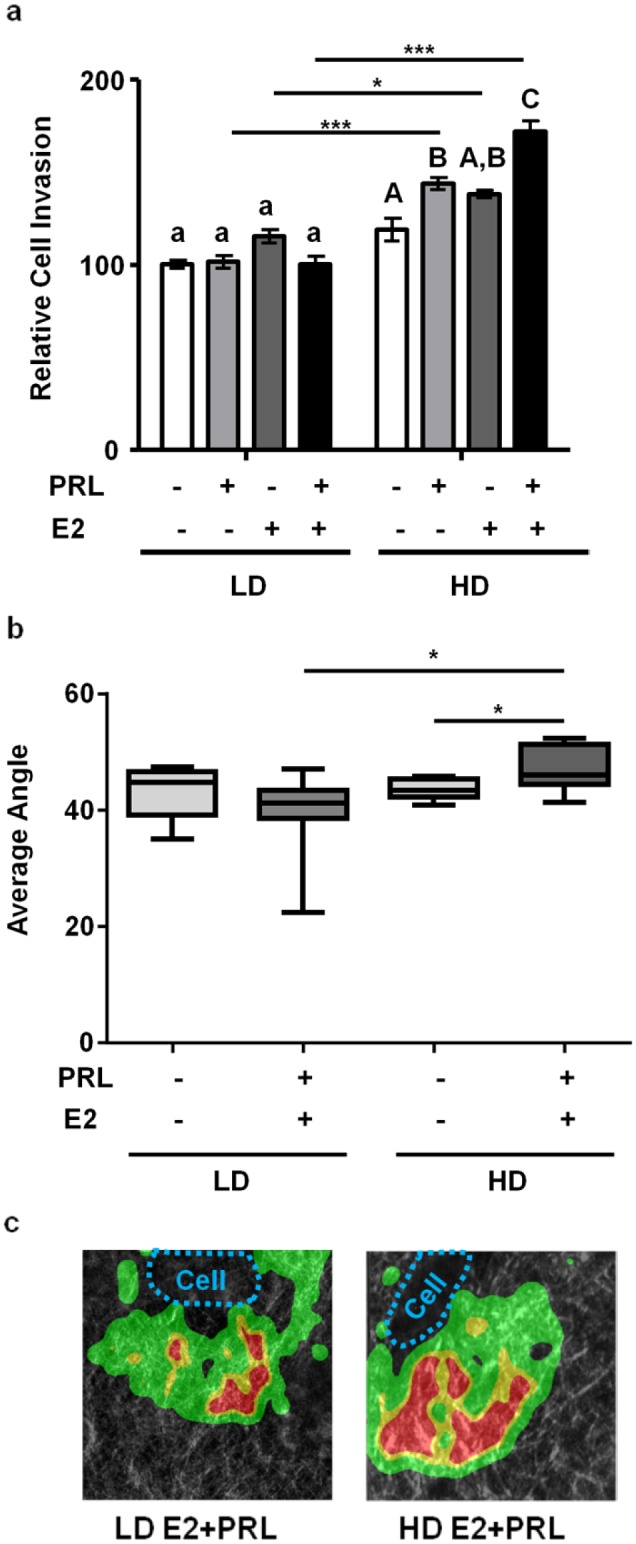
E2+PRL induces invasion only in high density/ stiff collagen matrices. **a)** MCF-7 cells were cultured in LD or HD collagen on 8µm transwell inserts, treated for 24hr with EtOH vehicle ± 2nM E2, ± 8nM PRL as indicated, and transversed cells were counted. n = 3. Asterisks indicate significant differences between the same treatments in different densities, *p<0.05, ***p<0.001. Different letters represent significant differences (p<0.05) between treatments within each matrix density condition. **b)** MCF-7 cells were cultured in LD or HD collagen and treated +/- 1nM E2, ± 4nM PRL for 72h. SHG images were collected and analyzed as described in the Materials and Methods. n = 7–8, *p<0.05. c) Representative heat maps of angles of collagen fibers relative to boundaries of cells cultured in low and high matrix densities co-treated with estrogen and PRL. Clear (black) areas represent 0–15 degrees; green, 15–45 degrees; yellow, 45–60 degrees; red, 60–90 degrees.

Cancer progression is characterized by local invasion of tumor epithelia, which may lead to distant metastases [64]. In order to determine the effect of matrix density on E2 and PRL crosstalk on invasiveness, MCF-7 cells were cultured in low density or high density collagen-I using transwell inserts and treated for 24h. Hormones failed to induce invasion in compliant matrices. However, in stiff matrices, PRL alone significantly increased invasion, and E2+PRL further augmented invasion compared to either hormone alone (p<0.05) and to treated compliant cultures (p<0.001) (Fig. 7a).

One of the hallmarks of aggressive clinical ERα+ breast tumors is modification of the alignment of collagen fibers, so that they become more perpendicular to the cell surface [10]. We utilized SHG imaging to examine the effect of hormones on the orientation of collagen fibers surrounding MCF-7 cells in compliant and stiff matrices. Co-treatment with E2 and PRL of cells in high density matrices induced a modest, yet significant increase in the mean angles of all collagen fibers relative to cell surfaces compared to vehicle-treated and hormone-treated low density collagen I cultures (p<0.05) (Fig. 7b, c). Together, these results indicate that E2 and PRL interact to drive cell invasion and matrix reorganization only in a stiff ECM environment.

## Discussion

**Figure 8:**
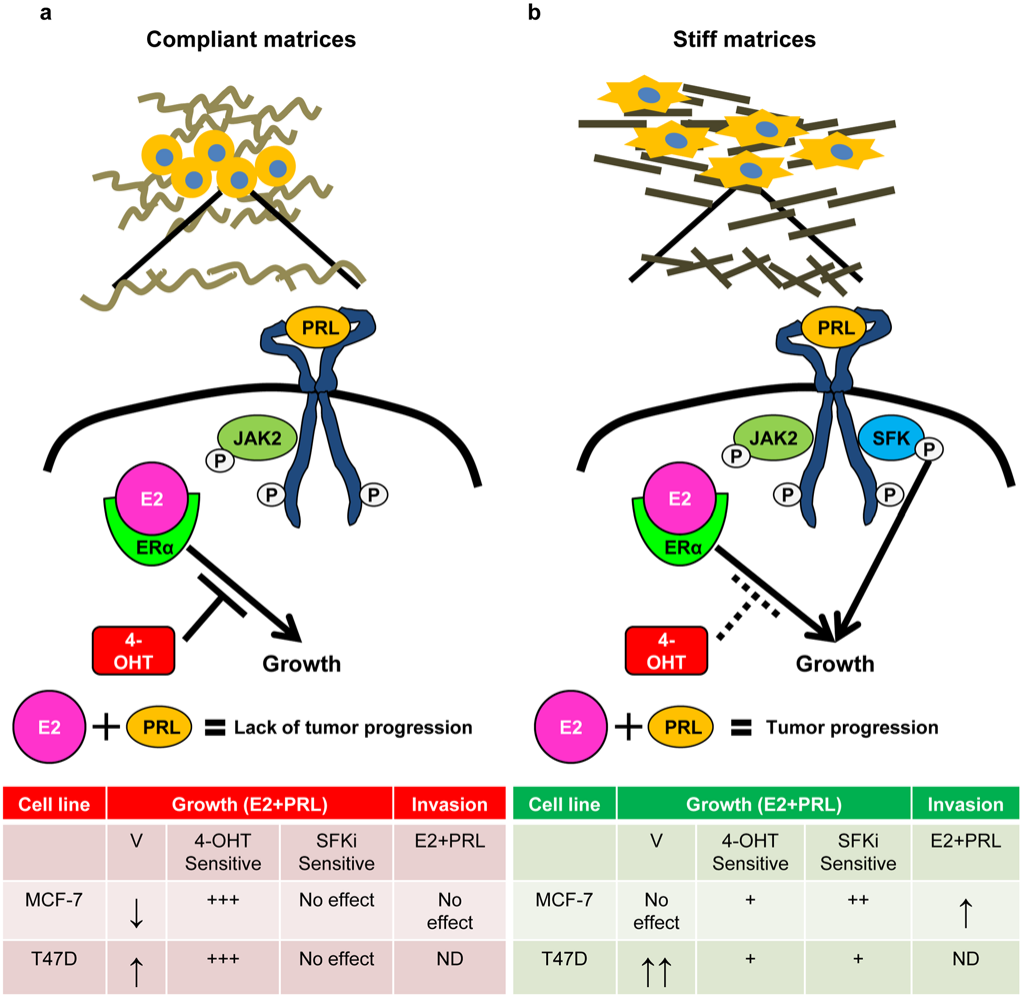
Summary of the effects of differences in collagen matrix density on estrogen and PRL crosstalk in breast cancer cells. **a)** Low density/ compliant collagen matrices permit modest cooperation between E2 and PRL to growth of T47D cells and inhibit E2-induced growth in MCF-7 cells. E2-PRL induced growth is inhibited by 4-OHT, but not SFK inhibitors in both cell lines. Moreover, MCF-7 cells do not invade in response to hormones. These results indicate low density matrices do not allow estrogen and PRL crosstalk to stimulate tumor progression. **b)** High density/ stiff collagen matrices permit E2-PRL to enhance growth, reduce 4-OHT sensitivity, and drive invasion of MCF7 cells. SFKs are key regulators of the growth response in both cell lines only in stiff collagen matrices. Together, these results indicate that stiff matrices cooperate with E2-PRL crosstalk to fuel processes leading to tumor progression. V = vehicle, ND = not determined; ↑, + indicate relative strength of response.

Characteristics of the ECM increasingly are recognized as critical players in the progression of breast cancer. The desmoplastic response increases the stiffness of the matrix, which is implicated in metastatic progression [8, 12]. Tumor cells that invade away from the primary tumor encounter collagen-I as the major component of the ECM, and the orientation of these fibers provides physical highways for invasion [10, 65]. Although both elevated PRL levels and E2 supplementation are associated with increased mammographic density [66, 67], the effect of these changes in the ECM on the actions of these hormones in ERα+ breast cancer is poorly understood. Here, we demonstrated that matrix density altered E2-induced transcriptional responses, but failed to affect estrogen-induced growth. In contrast, increased matrix density potently modulated the outcomes of PRL-estrogen crosstalk. In low density matrices, the net effect of their interaction on processes underlying tumor progression was slight or even inhibitory. However, high density/ stiff collagen matrices enhanced growth in response to both hormones together, even when effects of individual hormones were null or small, and permitted modest but significant PRL-estrogen induced growth in the presence of 4-OHT. Moreover, the PRL-estrogen combination drove invasion and matrix reorganization only in stiff collagen-I matrices. These findings indicate that a high density/ stiff matrix environment permits PRL-estrogen crosstalk to promote breast cancer progression by fueling tumor growth and invasion (Fig. 8). Further, they provide insight into a potential clinically relevant pathway for therapy resistance, and suggest a feed-forward loop between the ECM and pro-tumorigenic actions of these hormones.

Even though the ability of estrogen to activate a canonical ERE was reduced in high density/ stiff collagen-I matrices, the net effect on endogenous transcripts was gene and cell line specific. These observations indicate that the various estrogen-responsive enhancers present in the complex promoters of endogenous target genes are tuned to matrix density, which may alter the outcome of estrogen action on some aspects of tumor phenotype. However, estrogen-induced growth and its responsiveness to 4-OHT, proliferation and invasiveness were unaffected by matrix density.

Although interactions between the ECM and PRL/PRLR signals in breast cancer have been examined [39, 68, 69], the current studies revealed striking effects of the combination of PRL and estrogen in a high density/ stiff ECM that were not apparent with either hormone alone. This cooperation, which was prevented by matrices of normal physiological stiffness, underscores the potent synergy of these factors in ERα+/PRLR+ breast cancer. Our results show that the interplay of a high density/ stiff ECM environment and these mammotropic hormones can drive behaviors essential for tumor progression and treatment resistance, which would be especially harmful prior to diagnosis and treatment. Additionally, stiff matrices permitted PRL to increase estrogen-induced growth in the presence of 4-OHT. Although this growth in the presence of 4-OHT was modest (25% increase over 3 days in MCF-7 cells; 200% over 7 days in T47D cells in our studies), with time the increased accumulation of tumor cells *in vivo* would be substantial. Possible underlying mechanisms, such as increased 4-OHT agonist activity at ERα [4–6], or increased gpr30 activity [70] deserve future investigation. Others have reported that PRL also antagonizes traditional chemotherapies in breast cancer cells *in vitro* [58, 59, 71]. These observations provide an explanation for the clinical data linking PRL exposure to treatment resistance [17, 57, 72–74].

Interestingly, although the combination of PRL and estrogen augmented cell growth in high density/ stiff matrices in both cell lines, the underlying processes were different. However, despite these differences and the multiple reported mechanisms by which PRL and estrogen have been shown to interact, T47D and MCF-7 cells shared a common dependence on SFKs for PRL enhanced estrogen-induced growth in stiff matrices. We previously showed that a stiff collagen-I environment strengthens PRL signaling through the FAK-SFK-ERK1/2 cascade, associated with increased co-localization of FAK and PRLR [39]. In contrast, compliant collagen-I matrices favor PRL activation of the JAK2 effector, STAT5. FAK and SFKs are over-expressed in many aggressive breast cancers [13, 75–77], and these partners activate downstream mediators to drive invasion and proliferation [45, 78]. Our finding that SFKs are key mediators of PRL-enhanced estrogen-induced growth in stiff, but not in compliant matrices begins to reconcile the apparent conflict between evidence for PRL activity and activated STAT5 in outcomes of ERα+ breast cancer [17, 18, 36–38].

T47D and MCF-7 cells are two of the most studied ERα+/PRLR+ luminal breast cancer cell lines with respect to both estrogen and PRL actions [21–23, 39, 53, 79, 80]. While many reports document similarities in their responses to these hormones, they exhibit differences in the strength and time course of activation of down-stream signaling pathways [78], ligand regulation of PRLR expression [29, 81], and growth responses [82]. Moreover, they display striking differences in transcriptomes and resemblance to clinical breast cancer subtypes upon over expression of the PRL mediator, ELF5 [83]. Here we have shown that these cell lines also exhibit differences in the effect of matrix density on estrogen-regulated transcripts, and net outcomes of estrogen and PRL crosstalk on cell growth, relative effects on proliferation and survival, and dependence on JAK2 and SFK. Our results point to the importance of tumor cell context in determining hormonal responses, and the need to consider this facet of biology when experimentally modeling diverse luminal breast cancers.

In summary, we have shown that high density/ stiff collagen matrices enhance pro-tumorigenic crosstalk between estrogen and PRL in ERα+/PRLR+ luminal breast cancer cells. Our findings elucidate one mechanism by which the desmoplastic response around advancing ERα+ breast cancers may permit estrogen and PRL to accelerate tumor growth and invasion, and resist endocrine therapy. The differences in the two cell lines examined underscore the importance of examining multiple cell lines in preclinical studies. Together, these studies provide novel insights into the power of ECM stiffness to control the outcome of hormone signals in ERα+ breast cancers, and suggest new avenues for therapeutic approaches.

## Supporting Information

S1 Table qRT-PCR primers. Forward and reverse qRT-PCR primers utilized in this study.
(PDF)
