# Supplementary material for: Dense Collagen-I Matrices Enhance Pro-Tumorigenic Estrogen-Prolactin Crosstalk in MCF-7 and T47D Breast Cancer Cells
Source: PLoS One. 2015 Jan 21;10(1):e0116891. doi: 10.1371/journal.pone.0116891 (PMC4301649; doi:10.1371/journal.pone.0116891)
Supplement: S1 Table — Forward and reverse qRT-PCR primers utilized in this study. (PDF) [file pone.0116891.s001.pdf]

Supporting Information Table S1. qRT-PCR Primers

RT-PCR	
18S	F, 5’-CGC CGC TAG AGG TGA AAT TCT-3’<br>R, 5’-CGA ACC TCC GAC TTT CGT TCT-3’
TFF1	F, 5’-CGC CTT TGG AGC AGA GAG-3’<br>R, 5’-ACC ACA ATT CTG TCT TTC ACG-3’
CATD	F, 5’-GAC ACA GGC ACT TCC CTC AT-3’<br>R, 5’- GGA CAG CTT GTA GCC TTT GC-3’
PGR	F, 5’-CCT ATC CTG CCT CTC AAT CAC-3’<br>R, 5’-CCC GCC GTC GTA ACT TTC-3’
UGT2B15	F, 5’-GTG TTG GGA ATA TTA TGA CTA CAG TAA C-3’<br>R, 5’GGG TAT GTT AAA TAG TTC AGC CAG T-3’
